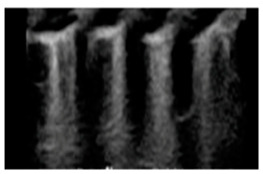# The Role of Lung Ultrasound Scan in Different Heart Failure Scenarios: Current Applications and Lacks of Evidences

**DOI:** 10.3390/diagnostics15010045

**Published:** 2024-12-28

**Authors:** Alessandro Campora, Matteo Beltrami, Anita Di Renzo, Alessia Petrini, Alberto Palazzuoli

**Affiliations:** 1 Department of Medical Biotechnologies, Division of Cardiology, University of Siena, Viale Bracci 1, 53100 Siena, Italy; alessandro.campora94@gmail.com (A.C.); anita.direnzo@unisi.it (A.D.R.); alessia.petrini@unisi.it (A.P.); 2 Arrhythmia and Electrophysiology Unit, Careggi University Hospital, 50134 Florence, Italy; beltrami.matteo1@gmail.com; 3 Cardiovascular Diseases Unit, Cardio Thoracic and Vascular Department, Le Scotte Hospital, University of Siena, 53100 Siena, Italy

**Keywords:** congestion, lung ultrasound, heart failure, respiratory diseases

## Abstract

Pulmonary congestion is a critical factor influencing the clinical presentation, therapeutic decisions, and outcomes of heart failure (HF) patients. Lung ultrasound (LUS) offers a simple, rapid, and accurate method for assessing pulmonary congestion, surpassing the diagnostic capabilities of traditional clinical evaluation and chest radiography. Due to the wide availability of ultrasound equipment, congestion can be evaluated in multiple settings, ranging from emergency departments to intensive care units, including outpatient settings. A combined cardiopulmonary imaging approach, integrating LUS with other imaging modalities, enhances congestion assessment in both acute and chronic HF. This comprehensive approach provides valuable insights for HF management and risk stratification. However, optimizing the utilization of LUS remains a challenge, as standardized imaging protocols and B-line thresholds may vary across different clinical scenarios and HF phenotypes. Despite the widespread use of LUS in various HF settings, physician adoption and interpretation of LUS findings remain suboptimal. This review aims to provide a practical and clinical overview of LUS in HF, guiding clinicians towards the correct application and interpretation of this valuable tool in diverse HF contexts.

## 1. Introduction

Pulmonary congestion is a serious condition associated with volume overload in heart failure (HF). Early detection of the signs and symptoms of pulmonary congestion and prompt initiation or accurate optimization of HF treatment are crucial in this clinical setting. Lung ultrasonography (LUS) has emerged as a valuable diagnostic tool for evaluating pulmonary congestion, allowing for the direct visualization of structural changes associated with congestion. This approach is particularly useful in patients with acute dyspnea and laboratory natriuretic peptide levels in the “grey zone”, leading to a more sensible assessment. Given the widespread availability of ultrasound devices, congestion can be evaluated in various settings, from inpatient to outpatient care, as well as across different ranges of disease severities [1]. LUS can also help differentiate HF from other conditions that may present with similar symptoms, such as pneumonia, acute respiratory distress syndrome (ARDS), and pneumothorax. Integrating LUS into the evaluation of pulmonary congestion in HF patients can improve clinical diagnosis, management, and therapeutic decisions, potentially reducing hospitalization rates and severe complications.

This review aims to provide a practical guide for clinicians on the use of LUS in patients with known or suspected HF, addressing the application of this tool in different HF phenotypes.

## 2. Congestion Occurrence

**Figure 1:**
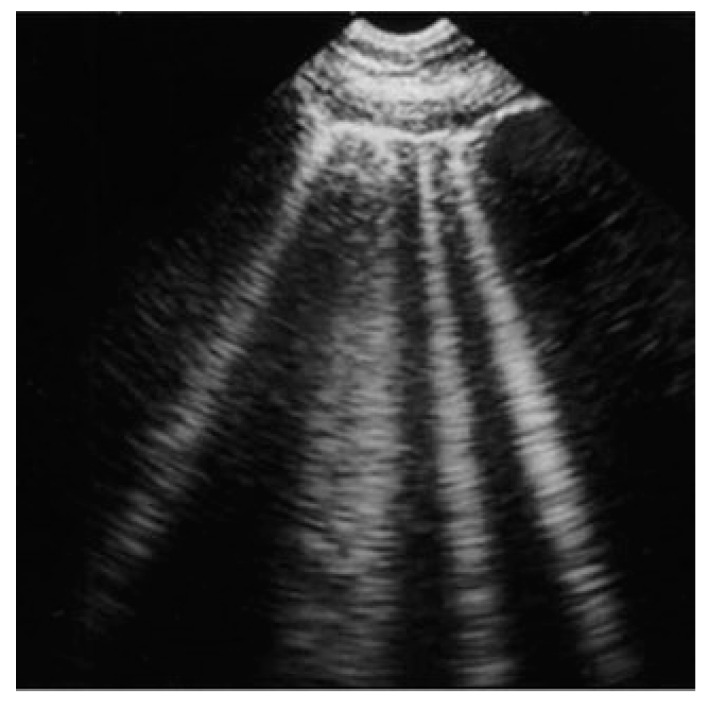
B-lines in lung ultrasound scan. B-lines, or ultrasound lung comets, can be visualized as discrete, laser-like, vertical hyperechoic reverberation artifacts, originating from the pleural line and extending to the image depth without intensity reduction. They move synchronously with lung sliding.

The congestion cascade typically begins with an increase in left ventricular filling pressures (hemodynamic congestion), which is transmitted backward to the left atrium and subsequently to the pulmonary circulation (pulmonary congestion). Pulmonary congestion specifically refers to the presence of extravascular fluid (extravascular lung water), which is visualized on LUS as discrete, laser-like, vertical hyperechoic reverberation artifacts originating from the pleural line, extending to the image depth without intensity reduction, and moving synchronously with lung sliding (B-lines, also known as ultrasound lung comets, Figure 1) [2,3]. Patients with similar left ventricular filling pressures can exhibit varying degrees of pulmonary congestion, ranging from a complete absence of extravascular lung water to alveolar lung edema. Numerous physiopathological mechanisms contribute to these differences, including the integrity of the alveolar-capillary membrane, the degree of systemic inflammation, the levels of hydrostatic and oncotic pressures, the rate of increase in left ventricular filling pressures, disease duration, and the efficiency of lymphatic drainage.

Two forms of pulmonary congestion exist: intravascular and interstitial. HF patients often present with a combined form of congestion [4,5]. These two types of congestion differ in their pathogenesis and onset mechanisms, yet both benefit from treatment with loop diuretics, which constitute the cornerstone of decongestant therapy. Loop diuretics reduce circulating volume, thereby addressing intravascular congestion with a lesser impact on interstitial fluid accumulation [6]. As for interstitial fluid accumulation, the loop diuretics may induce a decrease in blood osmolarity, which can initially limit fluid translocation from tissues to the intravascular space. In these patients, aquaretic drugs (such as vasopressin antagonists), acting by eliminating water and thus increasing blood osmolarity, may promote fluid translocation from tissues to the circulation, reducing tissue congestion [6].

## 3. Principle of LUS Scan

LUS is a readily available and reproducible diagnostic tool for cardiologists to evaluate HF patients during echocardiographic examinations. The principle underlying LUS evaluation relies on chest conformation, lung tissue composition, and the resulting acoustic interface between air and surrounding tissues. In normally aerated lungs, LUS examination reveals only the pleura as a horizontal line that moves with respiration (a finding known as “lung sliding”). When air content decreases due to interstitial or alveolar filling with transudate, exudate, and/or blood, the acoustic mismatch is reduced, allowing ultrasound to penetrate beneath the pleura, producing the vertical hyperechoic artifacts called B-lines. The lesser the volume occupied by air, the greater the number of B-lines observed. In the absence of air, the lung parenchyma appears as a solid organ with an echogenicity similar to the liver [7].

Typical B-lines associated with cardiogenic pulmonary congestion appear hyperechoic, thin, regular, and tend to be confluent and symmetrical. In contrast, non-cardiogenic B-lines are often more irregular, originating from the pleural line and appearing shorter and thicker.

The LUS technique involves positioning the echocardiographic transducer over the intercostal spaces, using either a longitudinal or transverse approach. The probe is then systematically moved across the patient’s chest following a simple scanning pattern.

**Figure 2:**
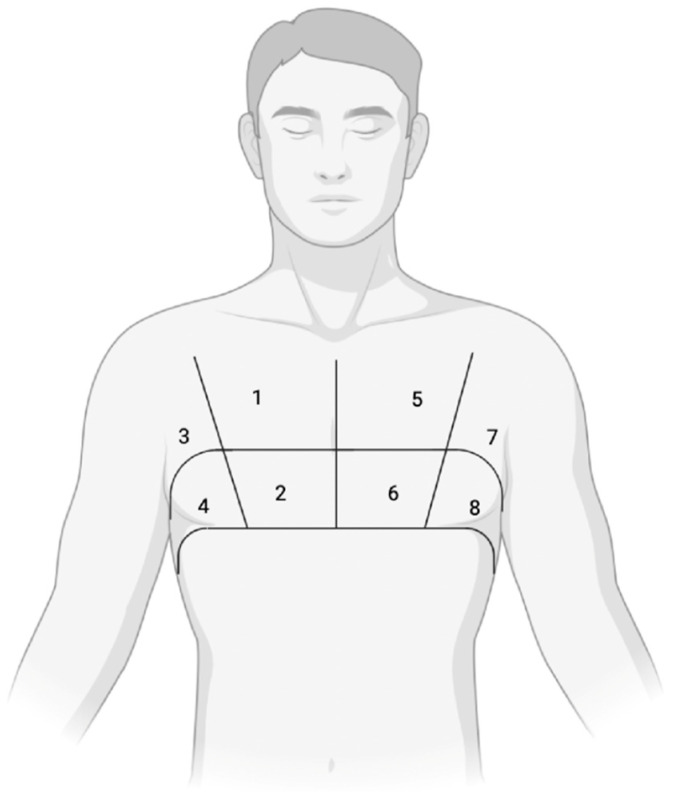
The simplified 8-zone protocol. This method divides each hemithorax into four zones, giving a total of eight zones for both lungs: upper anterior; lower anterior; upper lateral; lower lateral.

The simplified 8-zone protocol (Figure 2) is increasingly used in both clinical and research settings [8], typically taking around 5 min to performed. This approach has proven particularly useful in urgent conditions such as acute pulmonary edema. The eight zones are located in the anterior and lateral hemithorax, spanning from the parasternal line to the mid-axillary line, and from the second to the fifth intercostal space on the right side and the second to the fourth intercostal space on the left side. The lower intercostal spaces are excluded. A more comprehensive 28-zone scheme (16 zones on the right and 12 on the left) has been developed and may be useful for patient monitoring and prognosis assessment, particularly in stable outpatient settings [9].

Two primary approaches are used to quantify pulmonary congestion based on B-lines presence: scoring and counting. In the scoring approach, a zone is considered “positive” if it contains three or more B-lines. The counting approach involves counting B-lines within a single zone and then summing the number of positive zones. B-lines can be quantified by either counting them individually within a chest zone or, when they are confluent, estimating their number based on the percentage of the lung area they occupy below the pleural line. This estimation is typically conducted by dividing the percentage by 10. For example, if approximately 70% of the lung area below the pleural line is filled with B-lines, it would be counted as seven B-lines, with a maximum of ten per zone. The diagnostic power of B-lines has been extensively demonstrated in various studies [10].

In patients presenting to the emergency department with acute dyspnea [11], a cut-off value of ≥3 B-lines in at least 2 zones per hemithorax (of 6–8 evaluated zones total), in conjunction with the standard diagnostic framework, can identify patients with acute HF with higher sensitivity (94–97%) and specificity (96–97%) compared to physical examination, chest X-ray (CXR), and N-terminal pro b-type natriuretic peptide (NT-proBNP) (sensitivity 85%, specificity 89–90%). Additionally, LUS has shown to have prognostic implications for HF patients, both in inpatient and outpatient settings. Several studies have shown that persistent B-lines at discharge after hospitalization for acute HF predict a higher risk of HF exacerbation and rehospitalization at 3–6 months [12]. Similarly, outpatients with a high B-line count are at increased risk of HF exacerbation and hospitalization [13,14]. Therefore, especially in patients without overt clinical signs of congestion but with elevated left ventricular filling pressures and BNP levels, incorporating LUS into routine clinical practice may be crucial [7].

## 4. Role of LUS in the Emergency Department and Acute HF

The CXR is the traditional tool for diagnosing pulmonary congestion and is commonly used in HF assessment. Radiographic findings suggestive of HF include interstitial edema, pulmonary edema, and vascular congestion. However, HF can occur without radiographic signs of pulmonary congestion, particularly in patients with elevated natriuretic peptide levels, requiring further evaluation. In such cases, ESC guidelines recommend using LUS as a complementary diagnostic tool [15]. Therefore, emergency department physicians should not exclude patients without radiographic signs of congestion from the diagnosis of HF [16]. LUS is more sensitive than CXR in detecting pulmonary edema in patients with acute decompensated HF, making it a valuable addition to imaging techniques in the evaluation of dyspnoic patients at risk of HF [17]. Some of the main studies evaluating the usefulness of LUS in patients with acute HF are listed in Table 1.

Early identification of patients with congestion can lead to prompt hospitalization and improved patient management, with significant implications for reduced length of stay and fewer clinical complications. Combining LUS with NT-proBNP and other echocardiographic tools has been shown to reduce diagnostic delay, enabling earlier treatment initiation and improving overall outcomes [18]. The routine application of LUS may be particularly beneficial for patients with acute coronary syndrome, especially those with ST-segment elevation myocardial infarction (STEMI), who are at increased risk of developing pulmonary congestion. Accordingly, a recently published report suggests that a higher number of B-lines detected with LUS in these patients correlates with a greater risk of adverse events both during hospitalization and in long term outcome [19].

Since B-lines reflect changes in extravascular water, serial ultrasound assessments can be valuable in both acute and chronic settings, especially for patients with frequent hospitalizations. Rivas Lasarte et al. conducted a randomized trial comparing traditional and LUS-guided approaches, demonstrating that the latter facilitated more effective diuretic titration and a reduction in HF recurrences [20]. Notably, the reduction in B-line count from admission to discharge appears to be the most effective predictor of outcomes, despite variations in cut-off values reported in different studies [14].

Coiro et al. showed that a LUS finding of more than 30 B-lines after HF hospitalization was an independent predictor of poor outcomes, and that combining LUS with BNP and NYHA class identifies high-risk patients for rehospitalization [21]. In this study with a simplified model considering 4 windows per lung (simplified 8-zone protocol), congestion had been classified as mild (6–15 B-lines), moderate (16–30 B-lines), and severe (>30 B-lines). An average reduction of six B-lines from admission to discharge was associated with fewer adverse events. Palazzuoli et al. demonstrated that at discharge, cut-off values of 22 B-lines for HF with reduced ejection fraction (HFrEF) and 18 B-lines for HF with preserved ejection fraction (HFpEF) were associated with poor post-discharge outcomes [22]. Ruocco et al. demonstrated that in acute HF, limited reduction in both clinical congestion and B-lines number from admission to discharge was related to poor prognosis, irrespectively of HF subtype [23].

**Table 1:** Main studies evaluating the clinical usefulness of lung ultrasonography in patients with acute heart failure. AHF, Acute Heart Failure; CXR, Chest X-Ray; HF, Heart Failure; HFpEF, Heart Failure with Preserved Ejection Fraction; HFrEF, Heart Failure with Reduced Ejection Fraction; LUS, Lung Ultrasound.

Study	Results	References
**Coiro et al., 2015**	A B-line count ≥ 30 at discharge in patients hospitalized for AHF, is a strong predictor of all-cause death or HF hospitalization at 3 months	[21]
**Pivetta et al., 2015**	The LUS-implemented approach had a significantly higher accuracy (sensitivity, 97% [95% CI, 95–98.3%]; specificity, 97.4% [95% CI, 95.7–98.6%]) in differentiating AHF from noncardiac causes of acute dyspnea than the initial clinical workup, chest radiography alone, and natriuretic peptides.	[11]
**Platz et al., 2017**	In acute HF, ≥15 B-lines on 28-zone LUS at discharge identified patients at a more than five-fold risk for HF readmission or death	[5]
**Palazzuoli et al., 2018**	≥22 B-lines at discharge → cut-off for poor outcome at discharge in HFrEF. ≥18 B-lines at discharge → cut-off for poor outcome at discharge in HFpEF	[22]
**Maw et al., 2019**	LUS is more sensitive than CXR in detecting pulmonary edema in AHF. The relative sensitivity ratio of LUS, compared with CXR, was 1.2 (95% CI, 1.08–1.34; *p* < 0.001).	[17]
**Rivas-Lasarte et al., 2020**	The presence of subclinical pulmonary congestion at discharge (≥5 B-lines in LUS in absence of rales in the auscultation) was a risk factor for the occurrence of the primary combined outcome of rehospitalization or unexpected visit for HF worsening or death at 6- month follow-up (hazard ratio 2.63; 95% confidence interval: 1.08–6.41; *p* = 0.033).	[20]
**Palazzuoli et al., 2024**	Adding echocardiographic and LUS features of congestion to a model than included age, sex, systolic blood pressure, clinical congestion and natriuretic peptides in AHF patients, improved risk stratification at 90 and 180 days.	[18]
**Ruocco et al., 2024**	In AHF, the degree of congestion reduction assessed over the in-hospital stay period can stratify the subsequent event risk. Limited reduction in both clinical congestion and B-lines number are related to poor prognosis, irrespective of HF subtype.	[23]

## 5. Role of LUS in Outpatients

The typical evaluation of the degree of congestion in outpatients with HF relies on clinical conditions and physical examination, which have limited sensitivity in assessing pulmonary congestion. Early recognition of pulmonary congestion is crucial, as timely therapeutic interventions can reduce congestion, decrease hospitalization rates, and lower morbidity, mortality, and healthcare costs. This is particularly relevant considering that most patients with HFrEF are hospitalized more often due to pulmonary congestion rather than reduced cardiac output [24,25].

Congestion is a cardinal sign of HF, often manifesting as weight gain due to fluid retention. Notably, many patients in apparent good clinical condition, without peripheral edema or pulmonary rales, may exhibit subtle interstitial edema detectable by LUS. 

LUS is particularly valuable in the pre-decompensation stages of chronic HF and in patients with undiagnosed HF and vague symptoms. Primary care studies have demonstrated the utility of detecting B-lines in anterior, lateral, and posterior lung regions for identifying patients with HF, with a positive predictive value of 92% [8,10].

While natriuretic peptides correlate more with hemodynamic congestion, LUS provides information about pulmonary congestion. Both parameters are complementary tools that assess different pathophysiological mechanisms, offering valuable insights into the patient’s condition.

Miglioranza et al. [3] have shown that adding LUS to clinical evaluation provides prognostic value in outpatient settings, particularly in patients with moderate to severe systolic dysfunction. Specifically, the presence of more than 30 B-lines when performing a scan on 28 anterior and lateral chest regions has been associated with a high risk of hospitalization specifically for HF within the following 120 days. Conversely, patients with fewer than 15 B-lines had a low rate of hospitalization for HF and good overall survival. The main studies evaluating the usefulness of LUS in outpatients with chronic HF are listed in Table 2.

## 6. LUS in HFpEF, HFmrEF, HFrEF

The diagnostic and prognostic power of LUS appears similar for both HFpEF and HFrEF; LVEF and age did not influence the prognostic capacity of LUS in different clinical settings [13]. When analyzing the role of LUS in patients with HFpEF and HFrEF, it is noted that the number of B-lines is strongly associated with other measures of congestion. During hospitalization or at admission, B-lines correlate with BNP, atrial sizes, clinical signs of congestion, and measures of diastolic dysfunction, but do not predict patient outcomes. This holds true for both patients with HFrEF and HFpEF [29]. However, B-lines at discharge have been shown to predict clinical outcomes better than BNP in both HFpEF and HFrEF patients [22]. Additionally, the percentage reduction in B-lines has been associated with a lower congestion index at discharge, and residual congestion has been linked to a higher risk of rehospitalization or death [5,30]. Specifically, having more than 22 B-lines at discharge has been associated with negative outcomes [14,31]. LUS reveals a high prevalence of pulmonary congestion in patients with HFpEF and severe aortic stenosis undergoing Transcatheter Aortic Valve Implantation (TAVI), which is significantly reduced by the procedure. The assessment of pulmonary congestion pre-TAVI is an independent predictor of 1-year clinical outcome [32]. Moreover, exercise LUS showed good diagnostic value for HFpEF diagnosis regardless of different exercise protocols, particularly in patients with moderate-severe mitral regurgitation, adding peak or change B-lines on top of HFpEF scores and natriuretic peptides, significantly improving their diagnostic accuracy [33,34].

Ultrasound can be useful also to detect and quantify the presence of clinical or subclinical congestion in different organs across different HF phenotypes. Systemic venous congestion can be assessed by the evaluation of the hepatic, portal, and intrarenal vein flow patterns (also called venous excess ultrasonography or VEXUS). Venous congestion not only results in a dilated IVC, but also decreases hepatic, intestinal, and renal blood flow [35]. Notably, a holistic approach looking at lung and systemic congestion may be much more informative on congestion status location and severity, providing useful prognostic information. Patients with signs of ultrasound congestion (inferior vena cava of ≥21 mm, highest tertile of lung B-lines, discontinuous renal venous flow, and lower jugular vein distensibility ratio) identifies a high prevalence of sub-clinical congestion associated with poor outcomes [36]. Moreover, the combined evaluation of IVC diameter, jugular vein distensibility ratio, and B-lines provide additional value for predicting the development of HF in those patients at risk [37].

The combination of Doppler evaluation of renal, hepatic, and portal venous flow can provide additional value when seeking to detect and treat volume overload independently of HF phenotypes [38]. IVC and lung ultrasound-guided therapy significantly reduced subclinical congestion at discharge, reducing the composite outcome of readmission for HF, unplanned visit for worsening HF, or death at 90 days [39].

## 7. LUS and Body Mass Index (BMI)

Congestion is a common but often overlooked and undertreated condition in patients with acute HF, and it is associated with adverse outcomes [40,41]. The ultrasound patterns of congestion in HF patients can vary based on body mass index (BMI). Obesity can hinder the accuracy of LUS by interfering with the visualization of pleural lines and B-lines and while signs of systemic venous congestion, such as inferior vena cava dilation and hypo-collapsibility, and peripheral edema are equally prevalent across different BMI groups, obese patients exhibit lower natriuretic peptide levels and fewer B-lines on LUS compared to normal-weight individuals with similar levels of congestion [42].

Despite these challenges, LUS remains a valuable tool for assessing congestion in obese patients and potential applications include monitoring response to therapy, risk stratification, and guiding fluid management.

Obesity is frequently associated with dyspnea, even during mild exertion, and chronic peripheral edema. It can lead to sodium retention, increased intravascular volume, elevated blood pressure, and increased cardiac afterload, which can contribute to adverse cardiac remodeling and HF development [43]. Consequently, diagnosing HF in a dyspneic and/or edematous obese patient can be challenging. While a severely depressed left ventricular ejection fraction (LVEF) can clarify the diagnosis and guide treatment, a normal or near-normal LVEF and mildly elevated natriuretic peptides can leave clinicians uncertain about the underlying cause of symptoms.

In summary, both B-lines and BNP levels decrease with increasing BMI in HF patients, potentially limiting their ability to accurately predict congestion. However, recent studies have shown that B-lines decrease to a lesser extent than BNP levels, making them a more reliable marker for congestion assessment in obese patients. Brainin et al. and Palazzuoli et al. have demonstrated that B-lines decrease by 18% in chronic decompensated HF and by 12% in acute decompensated HF for every 5-unit increase in BMI, while BNP decreases by 28% for the same BMI increase. Therefore, LUS may be a valuable diagnostic and prognostic tool even in obese patients, helping clinicians differentiate between various causes of dyspnea, particularly in those with normal or mildly elevated NT-proBNP levels and preserved EF [42,44].

To address the impact of BMI on LUS accuracy, two main strategies can be considered: (1) BMI-based standardization: Standardizing the number of B-lines based on BMI can help account for the reduced visibility of B-lines in obese patients; (2) Serial monitoring: Monitoring changes in B-line count over time can provide valuable information about disease progression and treatment response, independent of baseline BMI.

## 8. Criticism of LUS

The limitations of LUS stem from its potential for false positives and the challenges associated with infrequent learning opportunities. B-lines can occur in various conditions, including non-cardiogenic pulmonary edema, pulmonary infections, and pulmonary interstitial fibrosis. However, specific sonographic characteristics, combined with patient history and clinical examination, can aid in differentiating between these etiologies [3,30,45,46]. Table 3 presents some of the characteristic LUS findings associated with specific clinical conditions.

There are instances where LUS can yield false-positive results, leading to misclassification of patients as having HF. This is particularly relevant in interstitial lung diseases, which can be caused by both infectious and fibrotic processes. Moreover, lung infections and HF-related edema often coexist, making it difficult to isolate the primary cause of symptoms.

Conversely, the absence of B-lines does not definitively rule out the diagnosis of HF. In individuals with high BMI or chest wall deformities, visualization of lung comets may be challenging. Therefore, a comprehensive assessment of both pulmonary and systemic congestion, along with careful correlation of imaging data with the clinical scenario, is essential. B-lines associated with cardiogenic pulmonary congestion exhibit specific characteristics, are more prominent in declivous lung regions (often accompanied by pleural effusion), and are commonly associated with cardiomegaly and other echocardiographic markers of cardiac dysfunction.

## 9. Future Applications of LUS

LUS has emerged as a valuable tool in HF patients due to its ability to provide a real-time, non-invasive, and bedside evaluation of pulmonary congestion. Its ability to detect changes in lung water content makes it a promising tool for various clinical applications. One key challenge in HF management is predicting decompensation. LUS shows promise in identifying patients at high risk of acute decompensation by detecting early changes in lung water content. Additionally, LUS can guide fluid management by providing real-time feedback on the effects of diuretic therapy, identifying patients who may require adjustments in their medication regimen. It should also investigate LUS as a guide for fluid management, specifically in optimizing diuretic therapy by monitoring the reduction of lung water content. Patients with acute HF managed with diuretics based on LUS findings of B-lines and pleural effusion showed a significant reduction in length of hospital stay and 90-day hospital readmissions [47]. Future research should further explore the potential of LUS as a prognostic tool, particularly its ability to identify patients at high risk of decompensation. Additionally, future research should explore the integration of LUS with artificial intelligence to enhance the accuracy and efficiency of its interpretation. Widespread adoption and physicians’ education on LUS are essential to realize its full potential. By expanding our understanding of LUS and integrating it into routine clinical practice, we can significantly improve the diagnosis, management, and prognosis of HF patients.

## 10. Conclusions

Lung ultrasound (LUS) has emerged as a valuable tool in the evaluation and management of heart failure (HF). Its ability to provide real-time, non-invasive assessment of pulmonary congestion offers several advantages over traditional methods. By detecting changes in lung water content, LUS can help identify patients at risk of decompensation, guide fluid management strategies, and assess treatment response.

However, the lack of standardization in LUS techniques, including the number of zones examined and the probe positioning, remains a significant challenge. This limits the comparability of results across different studies and hinders the development of evidence-based guidelines.

Future research should address these limitations by focusing on the standardization of LUS protocols and the development of objective criteria for image interpretation.

Additionally, investigating the role of LUS in identifying high-risk patients, optimizing diuretic therapy, and integrating artificial intelligence to enhance interpretation accuracy is crucial.

Widespread adoption of LUS, coupled with adequate training and education, is essential to fully realize its potential in improving the diagnosis, management, and outcomes of HF patients. By addressing the current limitations and promoting standardized practices, LUS can become an indispensable tool in the care of individuals with HF.

## Figures and Tables

**Table 2:** Main studies evaluating the clinical usefulness of lung ultrasonography in outpatients with chronic heart failure. AHF, Acute Heart Failure; HF, Heart Failure; LUS, Lung Ultrasound.

Study	Results	References
**Miglioranza et al., 2013**	A B-line ≥ 15 cutoff could be considered for a quick and reliable assessment of decompensation in outpatients with HF	[26]
**Platz et al., 2017**	in ambulatory patients with chronic HF, ≥3 B-lines on five- or eight-zone LUS marked those at a nearly four-fold risk for 6-month HF hospitalization or death.	[5]
**Miglioranza et al., 2017**	An outpatients B-lines number ≥ 30 (HR 8.62; 95%CI: 1.8–40.1; *p* = 0.006) identified a group of patients at high risk for acute pulmonary edema admission at 120 days, and was the strongest predictor of events compared to other established clinical, laboratory and instrumental findings.	[3]
**Rivas-Lasarte et al., 2019**	Tailored LUS-guided diuretic treatment of pulmonary congestion in this proof-of-concept study reduced the number of decompensations and improved walking capacity in patients with HF	[27]
**Marini et al., 2019**	LUS-guided management reduces hospitalization for AHF at mid-term follow-up in outpatients with chronic HF.	[28]

**Table 3:** Characteristic Lung Ultrasound patterns associated with specific clinical conditions. ARDS, Acute Respiratory Distress Syndrome; HF, Heart Failure.

Differential Diagnosis	LUS Pattern	Ecographic Image
**Acute and chronic HF**	Hyperechoic vertical lines extend from the pleural line (which is not thickened) and radiate towards the edge of the echocardiographic field.	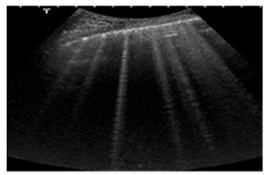
**Acute cardiogenic pulmonary oedema**	Number of hyperechoic vertical lines increased, extending from the pleural line and radiating to the edge of the ultrasound field, associated with pleural effusion (without thickening of the pleural line).	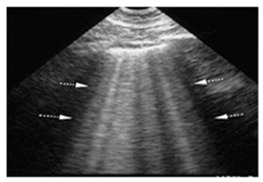
**ARDS**	“Multiple non-homogeneous B-lines with a non-gravity-dependent distribution, potentially coexisting with spared areas, along with pleural thickening, reduced or absent lung sliding, and subpleural or trans-lobar consolidations.”	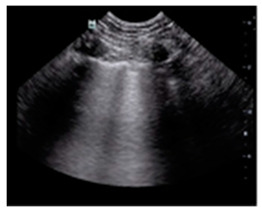
**Pneumonia/pulmonary consolidation**	“Lung consolidation occurs due to a significant loss of aeration. It appears as a tissue-like echotexture, with a superficial boundary at the pleural line and a deep, irregular boundary with the aerated lung, known as the ’shred sign.’”	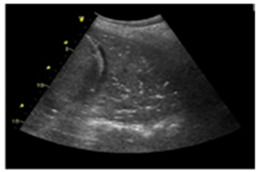
**Interstitial lung disease**	“Confluent B-lines with discontinuity of the pleural line”